# Comparing performance between clinics of an embryo evaluation algorithm based on time-lapse images and machine learning

**DOI:** 10.1007/s10815-023-02871-3

**Published:** 2023-07-10

**Authors:** Martin N. Johansen, Erik T. Parner, Mikkel F. Kragh, Keiichi Kato, Satoshi Ueno, Stefan Palm, Manuel Kernbach, Başak Balaban, İpek Keleş, Anette V. Gabrielsen, Lea H. Iversen, Jørgen Berntsen

**Affiliations:** 1 Vitrolife A/S, Jens Juuls Vej 18-20, 8260 Viby J, Denmark; 2 Section for Biostatistics, Department of Public Health, Aarhus University, Aarhus, Denmark; 3 The AI Lab Aps, Aarhus, Denmark; 4 Kato Ladies Clinic, Tokyo, Japan; 5 MVZ PAN Institut, Cologne, Germany; 6 American Hospital, Istanbul, Turkey; 7 Koc University Hospital, Istanbul, Turkey; 8 Fertility Clinic, Horsens Regional Hospital, Horsens, Denmark

**Keywords:** Embryo selection, Time-lapse, Artificial intelligence, Model performance

## Abstract

**Purpose:**

This article aims to assess how differences in maternal age distributions between IVF clinics affect the performance of an artificial intelligence model for embryo viability prediction and proposes a method to account for such differences.

**Methods:**

Using retrospectively collected data from 4805 fresh and frozen single blastocyst transfers of embryos incubated for 5 to 6 days, the discriminative performance was assessed based on fetal heartbeat outcomes. The data was collected from 4 clinics, and the discrimination was measured in terms of the area under ROC curves (AUC) for each clinic. To account for the different age distributions between clinics, a method for age-standardizing the AUCs was developed in which the clinic-specific AUCs were standardized using weights for each embryo according to the relative frequency of the maternal age in the relevant clinic compared to the age distribution in a common reference population.

**Results:**

There was substantial variation in the clinic-specific AUCs with estimates ranging from 0.58 to 0.69 before standardization. The age-standardization of the AUCs reduced the between-clinic variance by 16%. Most notably, three of the clinics had quite similar AUCs after standardization, while the last clinic had a markedly lower AUC both with and without standardization.

**Conclusion:**

The method of using age-standardization of the AUCs that is proposed in this article mitigates some of the variability between clinics. This enables a comparison of clinic-specific AUCs where the difference in age distributions is accounted for.

## Introduction

Evaluation of the embryonic potential for implantation and successful pregnancy has always been an important step during in vitro fertilization (IVF) treatments. Previously, these evaluations were based on manual assessments of static embryo morphology observed in a microscope [1] or morphokinetics observed using time-lapse equipment [2]. However, these evaluations are both time-consuming and prone to inter- and intra-observer variability [3]. The introduction of deep learning-based AI for embryo selection has enabled automated evaluations only based on images [4–6] or video sequences [7–9].

These AI models have the potential to improve, automate, and standardize the ranking and selection of embryos. To ensure that these promises can be achieved, it is important to apply transparent and standardized approaches for evaluating how well a model achieves its goal of predicting the implantation likelihood of an individual embryo. This ability is often referred to as *model performance* [10].

In practice, such AI models are used to rank embryos within a treatment cycle in order to choose the order of embryo transfer, where the embryos most likely to result in a positive outcome are chosen first. However, since we only know the clinical outcome of one or a few embryos within each cycle, the model performance is often evaluated on a population of transferred embryos.

The performance of predictive models can be divided into ranking and prediction performance, also known as model discrimination and model calibration [11, 12]. Model calibration is often assessed by comparing the predicted and achieved rates in subgroups with similar predictions. This is primarily a visual method, and in general, it is recommended to evaluate model calibration using plots rather than combined statistics [13]. Model discrimination is most commonly evaluated through the use of receiver operating characteristic curves (ROC curves) and the area under this curve (AUC). This measure, which is equivalent to Harrell’s concordance ($c$) index [14], evaluates the probability of the model correctly identifying the positive embryo in a randomly chosen pair of transferred embryos with one positive and one negative outcome.

Studies on model discrimination with regard to predicting ongoing pregnancy based on embryo image data have reported a wide range of different AUC values for the same model [12]. However, the actual estimated performance might depend on factors like the test data (internal/external), laboratory practice, patient demographics, selection criteria, culture conditions, ploidy testing, donor source, and others [12]. For example, maternal age is a well-known covariate that affects not only the likelihood of a positive fetal heart beat but also the model discrimination with AUC values reported to vary between 0.66 and 0.76 for different age subgroups within a single clinic [15]. Thus, differences in covariate distributions between two populations can bias the comparison of the model’s performance, which may lead to incorrect conclusions.

Biases from factors not directly related to the image data can potentially be mitigated by explicitly accounting for those factors in the AI model. In example, several models use age in combination with static images or time-lapse sequences as input [4, 6, 16–18]. The inclusion of age will improve the prediction of the actual probability of a positive outcome (i.e., the model calibration) as well as the model discrimination on the overall population of transferred embryos. However, no study has shown that including age as input also improves the model’s ability to select the most viable embryo within a treatment cycle where the age is constant. A simple and explicit approach for addressing the effect of a covariate on a measure of performance is to restrict the analysis to subgroups with similar values of the relevant covariate, for example, age. However, this might result in uncertain performance estimates due to the smaller sample size in the subgroups. Another approach is to summarize the performance overall using a *covariate-adjusted* ROC curve [19]. This approach generates a weighted average of covariate-specific ROC curves, and the AUC of this can then be calculated as a measure of covariate-adjusted model discrimination performance.

Within the methodological field of epidemiology, comparisons of measures of, e.g., incidence rates across heterogeneous subgroups are often done by using *direct standardization* where covariate-specific estimates within each subgroup are weighted according to the relative occurrence of the covariate value in similar subgroups of a common reference population [20, 21].

In this study, we will use a standardization procedure to evaluate the performance of the iDAScore v1.0 model [7, 22], which is a deep-learning model for embryo selection based on a 3D convolutional network that aims to predict fetal heart beat. The model is only trained on time-lapse sequences as input and thus does not use any patient- or clinic-related inputs. Several studies have shown that the iDAScore model correlates with morphology, morphokinetics, euploidy, fetal heart beat, and live birth outcomes [15, 23–26]. In the evaluation of model performance, we will consider model performance at the population level.

The aim of this study is to perform a comparison of the clinic-specific performance of an embryo selection AI model, accounting for the differences in age distributions between clinics.

## Materials and methods

### Study design and participants

The main study population, which we used to evaluate clinic-specific performance, comprises of data from four fertility clinics collected between 2013 and 2022. We only included transferred embryos incubated for 5 to 6 days with known maternal age and fetal heart beat outcome. Furthermore, we restricted to autologous oocytes, single-transfer embryos with maternal age between 21 and 44 years. The data set was not part of the training data for the iDAScore v1.0 model (Vitrolife A/S, Aarhus, Denmark), and hence, we refer to it as the *external population*. All data used in this study were retrospective and provided in an anonymized format. In Denmark, the study described was deemed exempt from notification to the National Committee on Health Research Ethics according to the Act on Research Ethics Review of Health Research Projects (consolidation act no. 1338 of September 1, 2020).

**Fig. 1:**
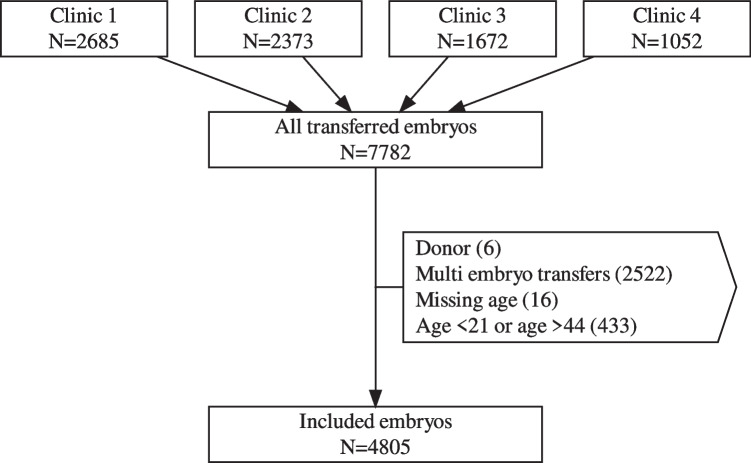
Flow chart of the external population containing data from four fertility clinics

The flow chart in Fig. 1 shows an overview of the exclusions in the external population.

We defined a reference age population to be used in the standardization based on the independent internal test data set that was held out during the training of iDAScore v1.0 [7]. We applied the same inclusion criteria for this population as defined for the external population.

The clinical endpoint of the study was defined as the presence of a fetal heart beat at 6–8 weeks after embryo transfer, and we distinguish between fetal heart beat positive (FH +) and fetal heart beat negative (FH-) outcomes.

### Statistical methods

We first visualized the age distributions and age-specific performance within each clinic. We estimated age distributions using a Gaussian smoothing kernel. The following describes the statistical methods used for assessing and comparing the clinic-specific discriminatory performance of the AI model accounting for the differences in age distribution between the clinics. We will use the term *age-standardized* to indicate that a performance measure accounts for age.

Neither the ROC curve nor the AUC measure can be standardized directly in order to account for differences in age distribution when comparing results between clinics, so the methods we applied are based on a ROC curve calculated on weighted data [27] in each clinic. We used the area under these curves to evaluate clinic-specific model performance. Since a ROC curve can be constructed from the sensitivity and specificity, the weights were applied in the calculation of these measures. This gives rise to a *weighted ROC* (WROC) curve, the area under which can be calculated and interpreted as a weighted, or *standardized*, AUC.

More specifically, since the sensitivity is based only on embryos with a positive outcome, for an FH + embryo, the weight is defined as the ratio between the age density in the positive subgroups of the reference and the clinic-specific populations evaluated at the maternal age. Similarly, the weight of an FH- embryo is the ratio between the age densities in the negative subgroups. This facilitates calculating weighted sensitivity and specificity values and thereby calculating the age-standardized AUC for each clinic. Please see Appendix 1 for more details on the weighting. We calculated clinic-specific AUCs based on both conventional ROC and WROC (AUC_ROC_ and AUC_WROC_) with 95% confidence intervals (CIs) estimated by bootstrap percentiles from 10,000 repetitions. We assessed the difference in the amount of variation between clinics by calculating the heterogeneity statistic, ${I}^{2}$, of both ${\mathrm{AUC}}_{\mathrm{ROC}}$ and ${\mathrm{AUC}}_{\mathrm{WROC}}$ estimates, separately, as well as the between-clinic variances, ${\tau }_{\mathrm{ROC}}^{2}$ and ${\tau }_{\mathrm{WROC}}^{2}$, as defined in a random effects meta-analysis [28, 29] using the DerSimonian and Laird method [30].

We also estimated the age-specific ROC curves using a semi-parametric approach [31] and bootstrap confidence intervals.

#### Software

All analyses were performed using R software [32] version 4.2.0, where we used the R packages WeightedROC [33] for estimation of weighted ROC curves and meta [34] for assessing between-clinic variation.

## Results

Table 1 shows the basic descriptive characteristics of the 4805 transferred embryos from 4086 treatments in the external and the reference populations. The four clinics contributing to the external population have FH + success rates ranging from 25.9 to 48.7%. It is apparent from the additional characteristics in Table 1 that the clinics adhere to quite different protocols. The table shows that the maternal age distributions differed between the clinics, since the median ages were substantially different. This is of particular interest for our analysis, since we will be standardizing the AUCs according to the age distributions. There were also differences in the use of fresh vs. vitrified embryos as well as the choice of insemination method (IVF vs. ICSI).

**Table 1:** Descriptive statistics of the transferred embryos from the external clinics and the reference population

Characteristic	Clinic 1	Clinic 2	Clinic 3	Clinic 4	Reference
No. of embryos	780	1959	1662	404	666
No. of treatments	661	1751	1270	404	650
Maternal age^†^	35 (32–38)	40 (37–42)	33 (30–37)	32 (28–35)	38 (34–41)
iDAScore^†^	8.3 (7.0–9.0)	8.8 (7.3–9.3)	9.0 (8.4–9.3)	9.2 (8.6–9.4)	8.6 (7.4–9.1)
FH + ^‡^	202 (25.9)	646 (33.0)	810 (48.7)	183 (45.3)	212 (31.8)
Frozen^‡^	269 (34.5)	1916 (97.8)	791 (47.6)	0 (0.0)	469 (70.4)
Treatment type					
ICSI^‡^	566 (80.5)	1333 (70.8)	335 (31.1)	397 (100.0)	466 (72.5)
IVF^‡^	137 (19.5)	549 (29.2)	743 (68.9)	0 (0.0)	177 (27.5)

^†^Values are given as median (IQR)

^‡^Values are given as *N* (%)

*IQR* interquartile range (25th and 75th percentiles)

**Fig. 2:**
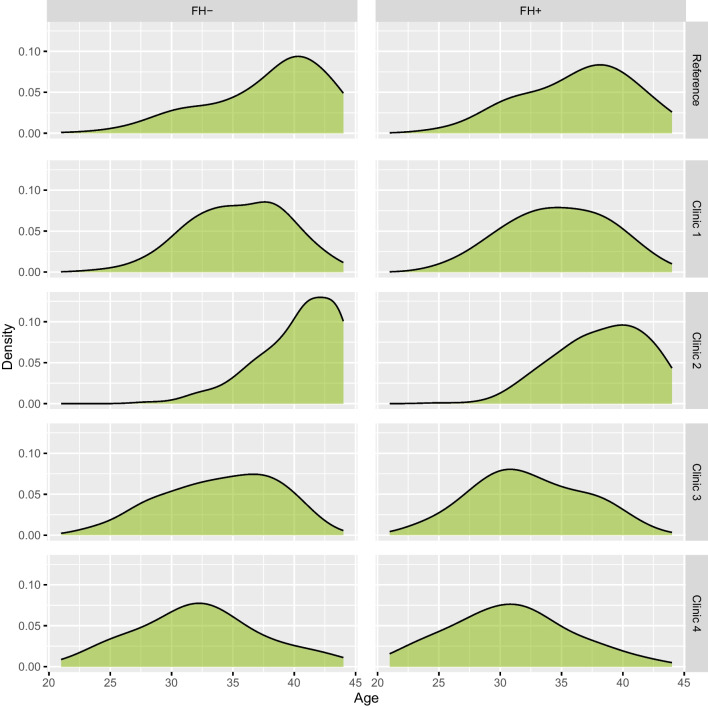
Clinic-specific maternal age distributions of the reference population and the external clinics for FH- and FH + embryos

To assess, in more detail, how the age distributions differed, we show the estimated density functions for each external clinic and the reference population in Fig. 2. From this plot, it is apparent that the clinics differed in both the variability and the shape of the maternal age distributions. It is also evident that there was some lack of overlap in the lower tails of the distributions, in particular for clinic 2.

**Fig. 3:**
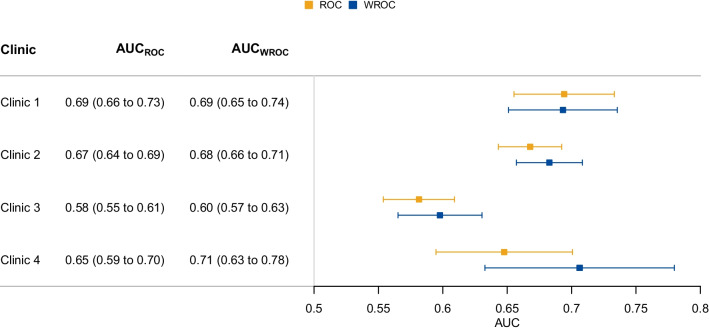
Estimates of clinic-specific ROC (orange color) and WROC (blue color) AUCs with 95% CIs

The clinic-specific AUCs based on both the ROC and WROC methods along with their bootstrap CIs are shown by the squares and whiskers in the forest plot in Fig. 3. The ${\mathrm{AUC}}_{\mathrm{ROC}}$ estimates varied substantially from 0.58 to 0.69, although with quite wide CIs. Clinic 3 showed a clearly lower AUC than the other three, which had some overlap in the CIs. The heterogeneity of the ${\mathrm{AUC}}_{\mathrm{ROC}}$ estimates was very large with ${I}^{2}=0.90$.

The ${\mathrm{AUC}}_{\mathrm{WROC}}$ estimates resulting from age-standardizing to the reference population varied from 0.60 to 0.71. The ${\mathrm{AUC}}_{\mathrm{WROC}}$ estimates were all higher than the ${\mathrm{AUC}}_{\mathrm{ROC}}$ estimates except for one (clinic 1) where there was almost no difference. This was in accordance with the relatively similar age distributions observed for this clinic and the reference population in Fig. 2. Clinic 3 still has a markedly lower age-standardized AUC than the other three clinics, which have more similar AUC values after standardization. The ${I}^{2}$ value for the ${\mathrm{AUC}}_{\mathrm{WROC}}$ estimates was 0.85, somewhat lower than the 0.90 for the ${\mathrm{AUC}}_{\mathrm{ROC}}$ estimates, and the estimated between-clinic variances were ${\widehat{\tau }}_{\mathrm{ROC}}^{2}=0.0026$ and ${\widehat{\tau }}_{\mathrm{WROC}}^{2}=0.0022$, representing a 16% decrease.

The mean of the clinic-specific ${\mathrm{AUC}}_{\mathrm{ROC}}$ estimates was 0.65 (95% CI: 0.61 to 0.68), while the mean of the ${\mathrm{AUC}}_{\mathrm{WROC}}$ estimates was 0.67 (95% CI: 0.63 to 0.71), which was equal to the conventional ${\mathrm{AUC}}_{\mathrm{ROC}}$ of the reference population up to two decimal places. This comparison was only possible because we have access to both model predictions and outcome data for the reference population in this study.

**Fig. 4:**
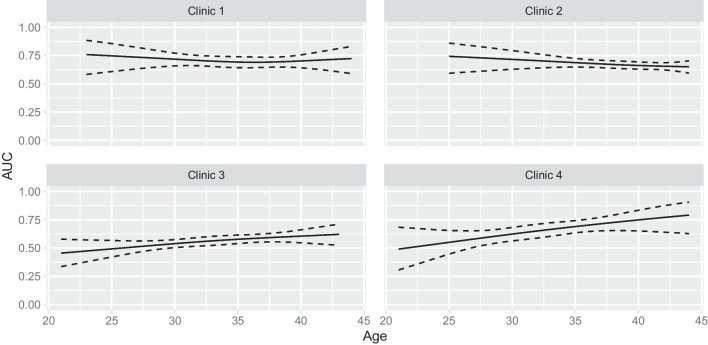
Estimated age-specific AUC for the external clinics. The solid line shows the estimated AUC values, while the dotted lines show the 95% confidence intervals

The relationship between model discrimination performance and maternal age within each clinic is illustrated in Fig. 4, which shows the age-specific AUC for each clinic. This figure shows that there was an association between maternal age and model performance but that maternal age affected model performance in quite different ways in the clinics, although the curves are subject to considerable uncertainty as reflected by the wide point-wise confidence intervals.

## Discussion

Comparisons of the performance of an AI model between different clinics are prone to suffer from biases introduced by differences in patient characteristics and other clinic-specific conditions. However, the evaluation of a model’s performance in different settings constitutes an important task for validation purposes as well as for obtaining knowledge about the variability of the performance.

In the existing literature, it is not uncommon to see direct comparisons of conventional ROC AUC between clinics. However, as we have argued, this does not give a complete and unbiased picture of the complex question of how model performance varies between clinics.

In this study, we applied a method based on weighted ROC to age-standardize the performance estimates between several different clinics thereby enabling a comparison that accounts for the inherent differences in maternal age between the clinics. Furthermore, we calculated the mean of the clinic-specific ${\mathrm{AUC}}_{\mathrm{WROC}}$ estimates as a summary measure of model performance across clinics. We found that the estimates of clinic-specific performance tended to vary less after applying standardization as compared to the conventional estimates, indicating that the standardization method reduces the between-clinic variability of the measured performance. It also turned out that in our data, the mean of the age-standardized AUC estimates was almost identical to the conventional AUC of the reference population, although this is not a result that could be expected to hold in general.

One obvious limitation of the approach we have used is that the adjusted value of each clinic-specific performance estimate depends on the particular age distribution in the reference population. On the other hand, this enables the comparison of performance in different settings whenever the age distribution is known in the comparative setting. In our case, all clinic-specific estimates increased as a result of the standardization, but this does not necessarily imply that the AUCs based on this approach are more meaningful than the unweighted estimates. However, it is arguably a more fair comparison of the model’s ability to rank embryos within treatment cycles where maternal age is constant, since it mitigates the effect of the maternal age differences between clinics. In fact, when we consider the age distributions and age-specific AUCs within clinics, it seems that for each clinic, the performance is lower in the age groups that are over-represented in that clinic compared to the reference.

Another limitation of the proposed method is that it relies on estimates of the age densities to account for the differences in age distributions, and, as such, the method might become unstable if there are maternal age ranges with low numbers of embryos. Finally, for this particular application of the method, the exclusion criteria that we have used in the study imply that our results do not necessarily apply to donor oocytes and to patients younger than 21 or older than 44 years.

The methods we propose in this paper are not restricted to the specific model (iDAScore) that we are evaluating. Numerous AI models, both commercial and academic, have been proposed for ranking of embryos. Some models are based solely on image data (single images or time-lapse), while others include other information relevant for implantation prediction. While the inclusion of patient characteristics, such as maternal age, in a model has the potential to improve the prediction of implantation likelihood, it might also hamper the generalizability of the model. Moreover, factors which are constant within a treatment do not have much potential for improving the ranking of embryos within the treatment. The iDAScore model is based on time-lapse images only, as this model is intended to give an objective and consistent evaluation and ranking of embryos within a treatment and not to predict the specific probability of implantation if the embryos are transferred.

Nor is the proposed method for comparing model performance between clinics restricted to standardizing on age distributions. The standardization can be done on any relevant characteristic as long as it is not used as input to the model that is being evaluated, and non-continuous variables could easily be handled as well. In fact, the methods could even be extended to account for multiple variables simultaneously by calculating the weights based on a multivariate probability distribution.

To facilitate the ability to objectively compare performance estimates from different settings would require the establishment of a common reference age distribution that could then be used as a standard within the field. Such a reference could, for example, be based on IVF data registries like the ESHRE data group [35].

For an individual clinic, it is of potential interest to compare the performance of a model to the performance of the same model in other clinics. But in such a comparison, it is essential to consider and account for the differences in patient characteristics. Therefore, comparisons should be performed on standardized rather than conventional AUC values. Furthermore, performance evaluation in different clinics is an integrated part of developing a model, and standardized AUCs provide a tool to obtain even better and more generalizable predictive algorithms within the field of embryo evaluation.

## Data Availability

The R code implementing the analyses in this study is available on request from the corresponding author. Data in this study is the property of the contributing clinics, and data requests can be directed to each clinic.

## Appendix 1. Methodological details

We consider a binary outcome, $D$, a continuous predictor, $X$, and a covariate, $Z$. In the following, we will show the calculations for a discrete variable $Z$, but the results can easily be transferred to the continuous case.

The unweighted ROC curve can be expressed in terms of the complementary conditional cumulative distribution functions for $X$, ${S}_{D=0}$ and ${S}_{D=1}$, as

$$\mathrm{ROC}(y)={S}_{D=1}\left({S}_{D=0}^{-1}(y)\right),$$

and the AUC is then defined as

$${\mathrm{AUC}}_{\mathrm{ROC}}={\int }_{0}^{1}\mathrm{ROC}(y)dy.$$

The weighted ROC curve ($\mathrm{WROC}$) is based on weighted sensitivity and specificity calculated for all possible thresholds.

### Weighted sensitivity and specificity

For a given threshold, $y$, we define the dichotomized prediction as ${T}_{X}=1(X>y)$ and consider the sensitivity,

$$\begin{array}{l}\mathrm{P}({T}_{X}=1|D=1)=\mathrm{E}(\mathrm{E}(1({T}_{X}=1)|Z,D=1)|D=1)\\ \;\;\;\;\;\;\;\;\;\;=\int \mathrm{E}(1({T}_{X}=1)|z,D=1){\mathrm{dP}}_{Z|D=1}(z)\\ \;\;\;\;\;\;\;\;\;\;\approx \sum\limits_{z}\frac{1}{{n}_{Z=z,D=1}}\sum\limits_{i}1({T}_{{X}_{i}}=1,{D}_{i}=1,{Z}_{i}=z)\frac{{n}_{Z=z,D=1}}{{n}_{D=1}}\\ \;\;\;\;\;\;\;\;\;\;=\frac{1}{{n}_{D=1}}\sum\limits_{i=1}^{{n}_{D=1}}1({T}_{{X}_{i}}=1,{D}_{i}=1).\end{array}$$

To compute the sensitivity under another $Z$ (age) distribution for the positives, ${\mathrm{P}}_{Z|D=1}^{*}$ say, write

$$\begin{array}{l}{\mathrm{P}}^{*}({T}_{X}=1|D=1)={\mathrm{E}}^{*}({\mathrm{E}}^{*}(1({T}_{X}=1)|Z,D=1)|D=1)\\ \;\;\;\;\;\;\;\;\;\;\;=\int \mathrm{E}(1({T}_{X}=1)|z,D=1){\mathrm{dP}}_{Z|D=1}^{*}(z)\\ \;\;\;\;\;\;\;\;\;\;\;=\int \mathrm{E}(1({T}_{X}=1)|z,D=1)\frac{{\mathrm{dP}}_{Z|D=1}^{*}}{{\mathrm{dP}}_{Z|D=1}}(z){\mathrm{dP}}_{Z|D=1}(z)\\ \;\;\;\;\;\;\;\;\;\;\;\;\mathrm{E}(1({T}_{X}=1)w(Z)|D=1),\end{array}$$

where $w(z)=\frac{{\mathrm{dP}}_{Z|D=1}^{*}}{{\mathrm{dP}}_{Z|D=1}}(z)$. This is estimated by

$${\widehat{\mathrm{P}}}^{*}({T}_{X}=1|D=1)=\frac{1}{{n}_{D=1}}\sum_{i=1}^{{n}_{D=1}}1({T}_{{X}_{i}}=1,{D}_{i}=1){w}_{i},$$

with ${w}_{i}=w({Z}_{i})$. Note that we used the distribution of $Z$ given $D=1$. In the specificity, the distribution of $Z$ given $D=0$ is used. The distribution of $Z$ often depends on $D=\mathrm{0,1}$ in the reference population. The package WeightedROC [17] defines the weights to be subject-specific and thereby allows for the possibility that the weights differ for the sensitivity and specificity. This is done in Keilwagen et al. [20]. Finally, note that we assumed that ${\mathrm{E}}^{*}(1(T=1)|Z,D=1)=\mathrm{E}(1(T=1)|Z,D=1)$.

### Weighted ROC and AUC

The weighted ROC curve can then be formed by plotting ${\mathrm{P}}^{*}({T}_{X}=1|D=1)$ against $(1-{\mathrm{P}}^{*}({T}_{X}=0|D=0))$ for the range of possible thresholds $y$. Denoting the complementary conditional cumulative distribution function for $X$ as

$${S}_{D=1}^{*}(y)={\mathrm{P}}^{*}(X>y|D=1)={\mathrm{P}}^{*}({T}_{X}=1|D=1)$$

and

$${S}_{D=0}^{*}(y)={\mathrm{P}}^{*}(X>y|D=0)=1-{\mathrm{P}}^{*}({T}_{X}=0|D=0),$$

we can express the weighted ROC curve as

$$\mathrm{WROC}(y)={S}_{D=1}^{*}({{S}_{D=0}^{*}}^{-1}(y)).$$

The area under the weighted ROC curve can then be defined as

$${\mathrm{AUC}}_{\mathrm{WROC}}={\int }_{0}^{1}\mathrm{WROC}(y)dy.$$
